# Wisconsin State Dental Society

**Published:** 1878-10

**Authors:** M. T. Moore


### WISCONSIN STATE DENTAL SOCIETY.

The seventh annual session of this body was held in Madison, July 9th, 10th and 11th, 1878.

The meetings were held in the Senate Chamber of the Capitol Building. Owing to the fact that the Society held no meeting in 1877, and that many members failed to receive due notice of the time, place and "Order of Business" of the session of 1878, the prevailing opinion among dentists, and especially members of the Society, in certain localities in the State was: that the session if not a complete failure, would at least be so slimly attended and poorly organized as to make it devoid of either pleasure or profit and a loss of time to any who might be present.

Those of the members who had sufficient interest and pride in the welfare of the organization—to the sustaining of which more than sixty dentists of this State have heretofore pledged their support—to attend the session recently held, have reason to feel fully satisfied and amply repaid.

During the session there were present sixty-two ladies and gentlemen, including prominent dentists from Illinois and Michigan.

TUESDAY, A. M.

The Society was called to order at ten o'clock and the time until noon was fully occupied in organizing and attending to usual routine business.

TUESDAY, P. M.

Most of the time of this session was occupied by report of special committee on Revision of Constitution and By-Laws. After careful consideration the report of the committee was adopted, and the Secretary was instructed to have printed a suitable number of copies for distribution and for the use of the Society.

The reports of the Secretary and Treasurer were read and referred to the Finance Committee. By vote the Society decided to hold no evening sessions.

WEDNESDAY, A. M.

The Society was called to order at nine o'clock, a. m. Dr. Chittenden introduced General George B. Smith, Mayor of the city, who most cordially welcomed the dentists of Wisconsin and adjoining States to the city of Madison, and in a pleasant and humorous manner rehearsed to those present the many privileges that he as Mayor was pleased to be able to extend to them. General Smith is a pleasant and graceful speaker, and his "Address of Welcome" was one of the features of the session.

ANNUAL ADDRESS.

Vice-president W. H. Chilson presided during the sessions and at the close of the welcome by Mayor Smith, he read the annual address, which was listened to with manifest interest, and for which he received a vote of thanks. So much of the address as related to the use of anæsthetics was on motion referred to a committee consisting of Drs. Stewart, Solliday and Babcock.

ESSAY.

Dr. C. A. Kitchen, of Rockford, Ill., read an essay entitled, "The Value of Dental Societies." The essayist gave, among other items of interest and importance, a history of dental societies, naming those first formed, and showing how they had benefited those who availed themselves of the advantages there offered. The essay was followed by discussion. Many present claimed that they owed their success entirely to what they had learned in dental societies. And all testifying to having been more than repaid for any expenditure of time and money they had incurred—in the assistance and information they had obtained at dental meetings.

WEDNESDAY, P. M.

The hours from two to five p. m. were devoted to clinics, with Drs. E. D. Swain, of Chicago, and B. G. Marcklein, of Milwaukee, as operators; the former using hand mallet and the latter the electric plugger. At five p. m. a business session was held, during which Milwaukee was selected as the next place of meeting.

WEDNESDAY EVENING.

The resident dentists invited all present to participate in an excursion on one of the lakes near the city. The arrangements for and pleasure of the excursion were such as to place the ladies and gentlemen invited under lasting obligations to those who provided the entertainment.

THURSDAY, A. M.

A committee consisting of Drs. Holbrook, Chittendon and Moore, to whom had been referred a "Bill to regulate the practice of Dentistry in the State of Wisconsin," reported that a bill had been presented at the last session of the Legislature, but owing to its being defective in one or two minor points it was decided not to take further action until the bill was properly framed. This was done, with the assistance of competent legal gentlemen, and the bill as corrected was adopted by the Society. On motion Drs. Chilson, Stillman and Chittenden were appointed a committee to secure the passage of the bill at the next session of the Legislature.

The committee to whom was referred that portion of the annual address relating to anæsthetics made their report, which was followed by an interesting and instructive discussion of the subject.

The Finance Committee submitted their report which showed that the Society is in a sound financial condition.

ESSAY.

Dr. E. D. Swain, of Chicago, read an essay, entitled "Diseased Conditions of the Soft Tissues of the Mouth; their Pathology and Treatment." The essay was followed by discussion which occupied the remainder of the morning session and was again before the Society at the afternoon session. Dr. Swain was repeatedly called upon and as often responded; giving much valuable information relative to the subject. The unanimous verdict was that the information received upon this subject alone amply repaid all present for the time and expense incurred in attending the session.

THURSDAY, P. M.

Most of the time was devoted to discussion of subjects as suggested and presented by the essay of Dr. Swain and the clinical operations of Wednesday.

During the session the following were elected and installed as officers of the Society for the ensuing year:

OFFICERS.

President, J. M. Baker, of Elkhorn; First Vice-president, C. E. Babcock, of Milwaukee; Second Vice-president, T. B. Fletcher, of Portage; Secretary, M. T. Moore, of La Crosse; Treasurer, L. C. Stewart, of Waupun.

President Baker appointed the following

COMMITTEES:

Executive—W. H. Chilson, Appleton; A. Solliday, Watertown; B. G. Marcklein, Milwaukee.

Membership—Wm. Decker, Oshkosh; R. S. Wells, Sparta; E. F. Long, Black River Falls.

Ethics—J. W. Cornelius, Madison; W. T. Hurd, Boscobel; J. S. Reynolds, Monroe.

Publication—M. T. Moore, La Crosse; A. H. Amos, Racine; E. Churchill, Columbus.

Finance—E. N. Clark, Beloit; R. W. Hurd, Madison; W. H. Ford, Beaver Dam.

Adjourned to meet in Milwaukee, July 8, 1879.

M. T. Moore, Secretary.

